# HTLV-1 Tax Stimulates Ubiquitin E3 Ligase, Ring Finger Protein 8, to Assemble Lysine 63-Linked Polyubiquitin Chains for TAK1 and IKK Activation

**DOI:** 10.1371/journal.ppat.1005102

**Published:** 2015-08-18

**Authors:** Yik-Khuan Ho, Huijun Zhi, Tara Bowlin, Batsukh Dorjbal, Subha Philip, Muhammad Atif Zahoor, Hsiu-Ming Shih, Oliver John Semmes, Brian Schaefer, J. N. Mark Glover, Chou-Zen Giam

**Affiliations:** 1 Department of Microbiology and Immunology, Uniformed Services University of the Health Sciences, Bethesda, Maryland, United States of America; 2 Institute of Biomedical Sciences, Academia Sinica, Taipei, Taiwan; 3 Department of Microbiology and Molecular Cell Biology, The Leroy T. Canoles Jr. Cancer Research Center, Eastern Virginia Medical School, Norfolk, Virginia, United States of America; 4 Department of Biochemistry, Faculty of Medicine and Dentistry, University of Alberta, Edmonton, Alberta, Canada; Duke University Medical Center, UNITED STATES

## Abstract

Human T lymphotropic virus type 1 (HTLV-1) trans-activator/oncoprotein, Tax, impacts a multitude of cellular processes, including I-κB kinase (IKK)/NF-κB signaling, DNA damage repair, and mitosis. These activities of Tax have been implicated in the development of adult T-cell leukemia (ATL) in HTLV-1-infected individuals, but the underlying mechanisms remain obscure. IKK and its upstream kinase, TGFβ-activated kinase 1 (TAK1), contain ubiquitin-binding subunits, NEMO and TAB2/3 respectively, which interact with K63-linked polyubiquitin (K63-pUb) chains. Recruitment to K63-pUb allows cross auto-phosphorylation and activation of TAK1 to occur, followed by TAK1-catalyzed IKK phosphorylation and activation. Using cytosolic extracts of HeLa and Jurkat T cells supplemented with purified proteins we have identified ubiquitin E3 ligase, ring finger protein 8 (RNF8), and E2 conjugating enzymes, Ubc13:Uev1A and Ubc13:Uev2, to be the cellular factors utilized by Tax for TAK1 and IKK activation. *In vitro*, the combination of Tax and RNF8 greatly stimulated TAK1, IKK, IκBα and JNK phosphorylation. *In vivo*, RNF8 over-expression augmented while RNF8 ablation drastically reduced canonical NF-κB activation by Tax. Activation of the non-canonical NF-κB pathway by Tax, however, is unaffected by the loss of RNF8. Using purified components, we further demonstrated biochemically that Tax greatly stimulated RNF8 and Ubc13:Uev1A/Uev2 to assemble long K63-pUb chains. Finally, co-transfection of Tax with increasing amounts of RNF8 greatly induced K63-pUb assembly in a dose-dependent manner. Thus, Tax targets RNF8 and Ubc13:Uev1A/Uev2 to promote the assembly of K63-pUb chains, which signal the activation of TAK1 and multiple downstream kinases including IKK and JNK. Because of the roles RNF8 and K63-pUb chains play in DNA damage repair and cytokinesis, this mechanism may also explain the genomic instability of HTLV-1-transformed T cells and ATL cells.

## Author Summary

Activation of the NF-κB family of transcription factors by the HTLV-1 oncoprotein, Tax, is causally linked to adult T cell leukemia (ATL) development in HTLV-1-infected individuals, but the underlying mechanisms are not fully understood. NF-κB activation requires the phosphorylation of its inhibitor, IκBα, by IκB kinase (IKK), which marks IκBα for degradation. In this study, we demonstrate that Tax inappropriately activates a ubiquitin E3 ligase, RNF8, and ubiquitin E2 conjugating enzymes, Ubc13:Uev1A/Uev2, to assemble long lysine 63-linked polyubiquitin (K63-pUb) chains, which function as signaling platforms for polyubiquitin-binding TGFβ-activated kinase 1 (TAK1) and IKK to congregate and become activated. Because TAK1 mediates the activation of multiple downstream signaling pathways, the mechanism described here can explain the complex effect of Tax on cell signaling. The major functions of RNF8 are to signal cellular DNA damage repair (DDR) and cell division by assembling K63-pUb chains at the site of DNA damage and cell cleavage. As such, the inappropriate activation of RNF8 and the over-abundance of K63-pUb chains in Tax-expressing cells may explain how Tax causes DNA damage and cell division defect.

## Introduction

Human T-lymphotropic virus type 1 (HTLV-1) is the etiological agent of adult T-cell leukemia/lymphoma (ATL) and HTLV-1-associated myelopathy/tropical spastic paraparesis (HAM/TSP). The HTLV-1 genome encodes a trans-activator/oncoprotein known as Tax, which is crucial for viral transcription and cellular transformation [1]. Tax constitutively activates IκB kinases, causing the phosphorylation and degradation of IκBα and activation of the canonical NF-κB pathway. It also up-regulates RelB and p100 (precursor of NF-κB2) expression and increases the phosphorylation and proteolytic processing of p100 to p52, thus activating the non-canonical NF-κB pathway [2–4]. IKK/NF-κB activation by Tax is causally linked to T-cell transformation and ATL development [5–9]. The mechanisms underlying Tax-induced IKK activation remains unclear, but require the regulatory subunit of IKK: NF-κB essential modulator (NEMO) and two IKK kinases: TGF-β-activated kinase 1 (TAK1) and NF-κB-inducing kinase (NIK) [10–12].

Polyubiquitin chain assembly is a post-translational modification by which protein-linked or unanchored polyubiquitin chains are synthesized stepwise via reactions requiring three classes of enzymes, E1 activating enzymes, E2 conjugating enzymes and E3 ligases. Lysine 48 (K48)-linked polyubiquitin targets proteins for proteasome-mediated degradation, while lysine 63 (K63)-linked and linear polyubiquitins are crucial for cytokine-mediated IKK/NF-κB activation and DNA damage repair (DDR) [13–15]. Upon engagement of receptors by cytokines such as TNFα and IL-1, members of the TNF receptor-associated factor (TRAF) family such as TRAF6, TRAF2/5, and cIAP1/2 become recruited to the receptors and function as E3 ligases to assemble free or protein-anchored K63-linked polyubiquitin (K63-pUb) chains. The IKK kinase, TGFβ-activated kinase 1 (TAK1), and IKK both contain ubiquitin-binding subunits (TAB2/3 and NEMO respectively) that facilitate their recruitment to K63-pUb chains where TAK1 undergoes auto-phosphorylation/activation. Activated TAK1 then phosphorylates/activates IKK in its vicinity. IKK in turn phosphorylates IκBα, targeting it for K48-linked polyubiquitination and proteasomal degradation, thereby activating the canonical NF-κB pathway. Interestingly, conjugation of Tax by K63-pUb has been reported to correlate with IKK activation, and requires an E2 enzyme known as Ubc13. Indeed, mouse embryo fibroblasts containing bi-allelic deletion of the Ubc13 gene are deficient in supporting Tax-mediated NF-κB activation [16]. IKK activation by Tax in cytosolic extract was also inhibited by the K63R mutant of ubiquitin [17]. These results support the notion that K63-pUb assembly plays an important role in Tax-mediated IKK/NF-κB activation.

The holo Ubc13-containing E2s that carry out K63-pUb chain assembly are heterodimers consisting of a catalytically active Ubc13 and a catalytically inactive E2 variant, Uev1A or Uev2 [18]. The Ubc13:Uev1A heterodimer has been shown to be essential for IKK/NF-κB activation [13], while the Ubc13:Uev2 heterodimer is thought to be involved in DDR [18,19]. Here we show that Ubc13:Uev1A (or Ubc13:Uev2) and the E3 ubiquitin ligase, ring finger protein 8 (RNF8) are crucial for Tax-mediated IKK activation *in vivo* and *in vitro*. The ablation or over-expression of RNF8 drastically reduces or augments canonical NF-κB activation by Tax. We demonstrate that Tax directly stimulates RNF8 and Ubc13:Uev1a (or Ubc13:Uev2) to assemble long and free K63-pUb chains *in vitro* and *in vivo*. The unanchored K63-pUb chains so assembled then activate TAK1, which in turn activates IKK and MKKs, and the downstream NF-κB and c-Jun N-terminal kinase (JNK) signaling pathways. In the presence of Tax, RNF8 is increasingly redirected from the nucleus to the cytoplasm. Because RNF8 is a key signaling molecule for DNA damage repair and cytokinesis; and because K63-pUb chains play many roles in cell signaling, the inappropriate activation of RNF8 and the over-abundance of K63-pUb chains produced in Tax-expressing cells activate multiple signaling pathways and likely also interfere with DDR and mitosis.

## Results

### The ubiquitin E2 conjugating enzyme, Ubc13:Uev1a, is required for Tax-mediated IKK activation *in vivo* and *in vitro*

**Fig 1:**
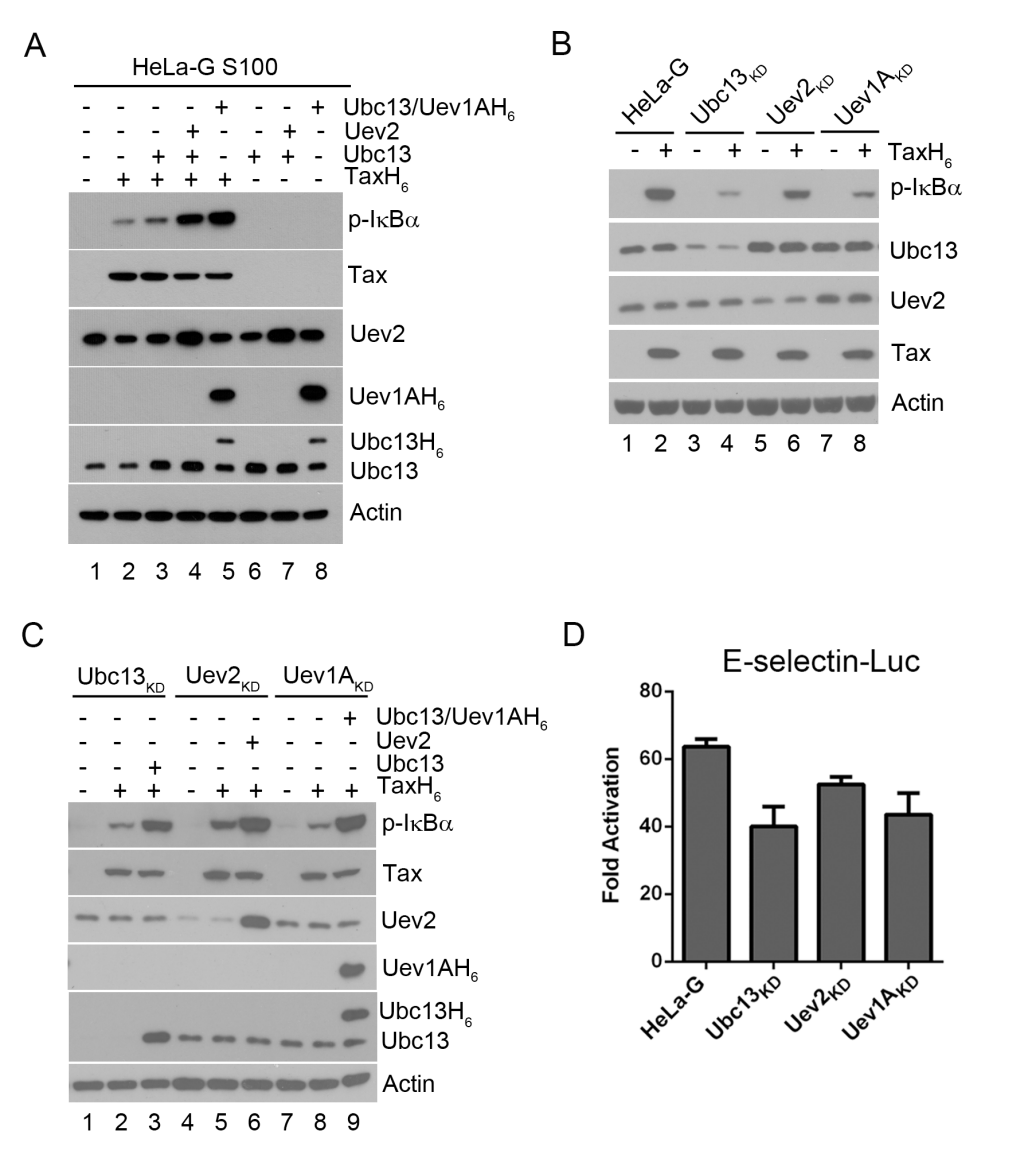
Ubc13-containing E2 enzymes are crucial for IKK activation by Tax *in vitro* and *in vivo*. **(A)** Ubc13-containing E2 conjugating enzyme complexes enhanced IKK activation by Tax in cell-free HeLa-G S100 extracts. The HeLa-G cytosolic S100 extract [17,20] was incubated with recombinant TaxH_6_ alone (lanes 2), Ubc13, Ubc13:Uev2, or Ubc13:Uev1AH_6_ with (lanes 4–5) or without TaxH_6_ (lanes 6–8) as indicated at 30°C for 1 hour. IKK activation was detected by immunoblot with anti-p-IκBα. Uev1A was detected using anti-poly-Histidine antibody. **(B & C)** IKK activation by Tax is reduced by the depletion of Ubc13, Uev1A, or Uev2, but restored by exogenously added Ubc13, Uev2 or Uev1A. (**B**) HeLa-G, Ubc13_KD_, Uev1A_KD_ and Uev2_KD_ S100 extracts were generated and incubated with (lanes 2, 4, 6, and 8) or without (lanes 1, 3, 5, and 7) recombinant TaxH_6_, or (**C**) in the absence of exogenous factors (lanes 1, 4, and 7), in the presence of Tax without (lanes 2, 5, and 8) or with Ubc13, Uev2, or Ubc13:Uev1A H_6_ (lanes 3, 6, and 9) as indicated at 30°C for 1 hour. Uev1A protein was not detectable in immunoblot, and Uev1A_KD_ is determined via mRNA levels shown in S1 Fig. **(D)** Tax-induced IKK activation is attenuated in HeLa-G cells knocked down for Ubc13, Uev2, or Uev1A expression. The Ubc13_KD_, Uev1A_KD_ and Uev2_KD_ cell clones were transiently co-transfected with E-Selectin-Luc with or without Tax (Materials and Methods). Firefly luciferase activity was measured at 48 hours post-transfection. Fold of activation was calculated as the ratio of luciferase activities in cells with Tax over those in cells without Tax.

To elucidate the mechanism(s) underlying Tax-driven IKK activation, we used a cell-free system [17,20] in which the cytosolic extract (S100) of HeLa-G (Fig 1A) cells was supplemented with purified hexa-histidine-tagged Tax derived from *E*. *coli*. In this system, the addition of Tax and ATP to HeLa-G S100 extract activated IKK, as indicated by the appearance of phospho-IκBα (p-IκBα) in immunoblots (Fig 1A, lane 2). Interestingly, addition of either Ubc13:Uev2 or Ubc13:Uev1A E2 conjugating enzyme complex into the S100 extract further stimulated Tax-dependent IKK activation as evidenced by the increased phosphorylation of IκBα (lanes 4 & 5), but had no effect in the absence of Tax (lanes 6–8). Importantly, the same results were observed in S100 extract prepared from Jurkat T cells with quantitative conversion of IκBα to p-IκBα in the presence of Tax (S2 Fig), and addition of other E2 conjugating enzymes, UbcH5b and UbcH5c, had no effect on Tax-mediated IKK activation (S2 Fig).

When S100 extract was prepared from HeLa-G cells depleted of Ubc13, Uev2, or Uev1A by shRNA-mediated knockdown, IKK activation by Tax *in vitro* was significantly diminished (Fig 1B p-IκBα blot in lane 2 vs lanes 4, 6, & 8). The addition of purified Ubc13, Uev2 or Uev1A to the respective depleted S100 lysates restored IKK activation by Tax (Fig 1C lanes 3 vs 2; 6 vs 5; 9 vs 8). The extent of NF-κB activation by Tax as measured by the E-selectin-Luc reporter assay was also reduced in HeLa-G cells deficient in Ubc13, Uev2, or Uev1A (Fig 1D). The moderate impact of these knockdowns is likely due to NF-κB activation contributed by residual Ubc13 E2 complexes as well as the non-canonical NF-κB pathway (see below). These results agree with previous studies showing that Ubc13 is critical for Tax-mediated IKK/NF-κB activation [16]. Tax is not an E3 ligase however. No ubiquitin chain assembly could be detected in reaction mixtures containing only Tax, Ubc13:Uev1A (E2), E1, Ub, and ATP (see below). This prompted us to consider the possibility that a cellular E3 ligase may be recruited by Tax for IKK activation.

### Tax recruits the ubiquitin E3 ligase RNF8 for IKK activation

**Fig 2:**
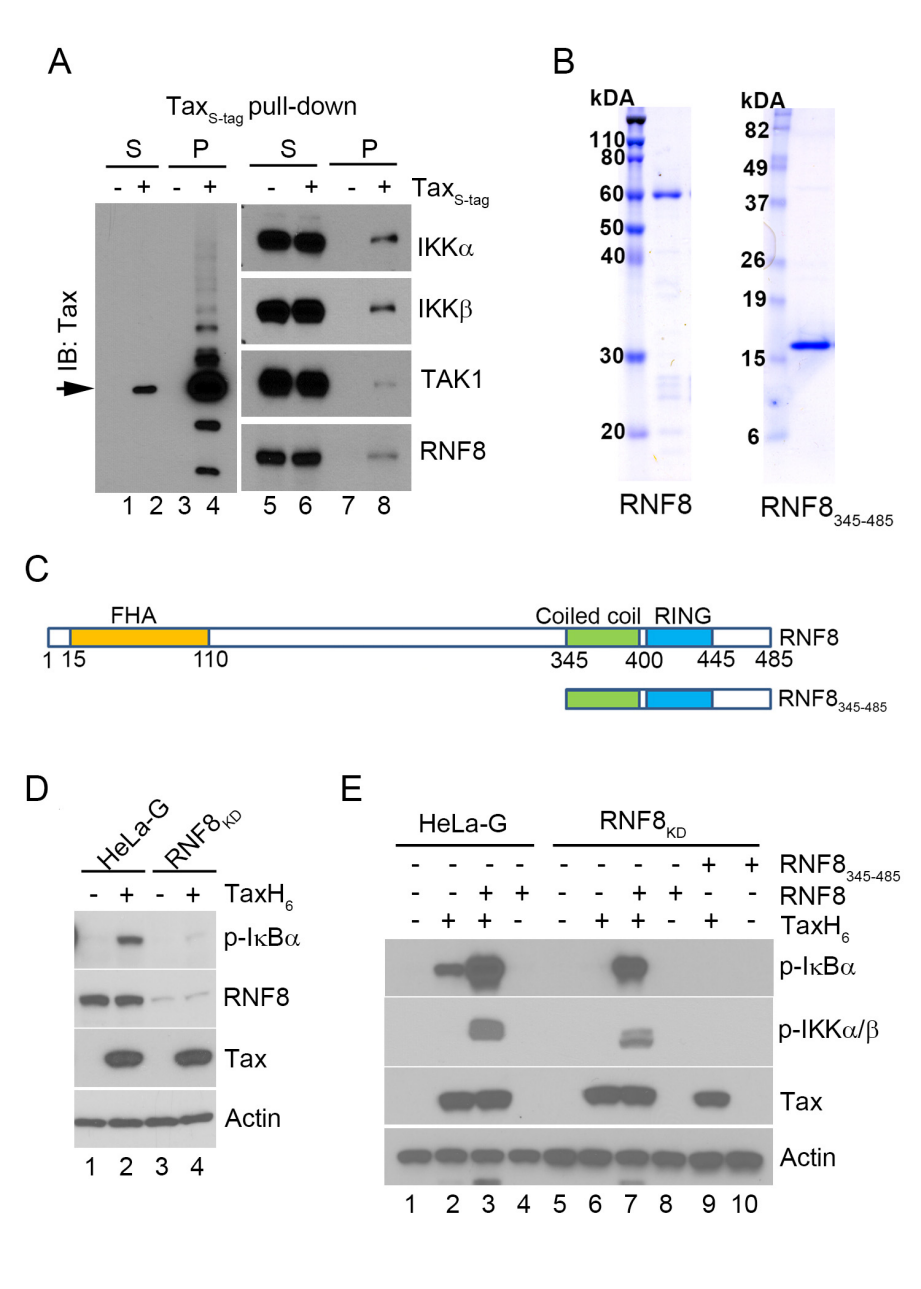
The E3 ubiquitin ligase, RNF8, supports IKK activation by Tax *in vi*tro. **(A)** RNF8 co-purifies with Tax_S-tag_ expressed in transiently transfected 293T cells. 293T cells that were transiently transfected with an S-tagged Tax expression plasmid were lysed and incubated with RNase S-agarose beads. The supernatant (S) and pull-down (P) fractions are immunoblotted with the indicated antibodies. **(B)** Commassie brilliant blue-stained full-length RNF8 and truncated RNF8_345–485_ purified from Sf9 insect cells and *E*. *coli* respectively. **(C)** Domain organization of RNF8 and a truncation mutant, RNF8_345–485_ **.** Amino acid residues that encompass the FHA domain, the coiled coil (CC) dimerization domain, and the RING finger domain are as indicated. **(D**) Depletion of RNF8 prevents IKK activation by Tax *in vitro*. HeLa-G (lanes 1 & 2) and RNF8_KD_ (lanes 3 & 4) S100 extracts were generated and incubated with or without recombinant TaxH_6_ as indicated at 30°C for 1 hour. IKK activation was detected by immunoblot with anti-p-IκBα. **(E)** Addition of RNF8 greatly enhances Tax-mediated IKK activation in S100 extracts from wild-type and RNF8_KD_ cells respectively. HeLa-G (lanes 1–4) and RNF8_KD_ (lanes 5–10) S100 extracts were incubated with recombinant TaxH_6_, RNF8 and/or RNF8_345-485_ as indicated at 30°C for 1 hour. Significant IKK activation was detected by immunoblot with anti-p-IκBα and anti-p-IKKα/β.

A search in the literature for E3 ligases that specifically utilize Ubc13 for ubiquitin chain assembly found C-terminus of HSC70-interacting protein (CHIP) [21], checkpoint with forkhead and RING finger domains (CHFR) [22], helicase-like transcription factor (HLTF) [23], RING finger protein 8 (RNF8) [24], TNF receptor-associated factor 2 (TRAF2), TRAF5, and TRAF6 (reviewed in [25]) to be of interest. Of these E3 ligases, RNF8 caught our attention. RNF8 is a E3 ligase that contains an Forkhead-Associated (FHA) domain for binding specific phospho-proteins, a coiled-coil region responsible for dimerization, and a RING domain for E2 binding and ubiquitin chain assembly (Fig 2C) [26–28]. RNF8 is important for DDR [29,30], centrosomal functions, and cytokinesis [31,32], all of which are known to be disrupted by Tax [33]. Furthermore, Tax is a dimer that interacts with and stabilizes the coiled-coil domains of dimeric bZip transcription factors CREB and ATF1 [34,35]. For these reasons, we tested RNF8 as a potential target of Tax. Indeed, RNF8 co-purifies with IKKα, IKKβ, TAK1, and Tax when S-peptide-tagged (S-tagged)-Tax is captured from transfected 293 cell lysate using RNase S-agarose beads (Fig 2A). Notably, a fraction of Tax was modified (Fig 2A left panel) with size increments consistent with polyubiquitination reported previously [16].

To investigate the role of RNF8 in Tax-induced IKK activation, glutathione S-transferase (GST)-RNF8 and GST-RNF8_345–485_ fusion proteins were purified and treated with 3C protease to remove GST, yielding highly purified full-length and truncated RNF8 (Fig 2B and 2C). As an indication that RNF8 is critical for IKK activation by Tax, the S100 extract prepared from HeLa-G cells depleted of RNF8 by shRNA-mediated knockdown (RNF8_KD_) was unable to support IKK activation by Tax (Fig 2D p-IκBα blot lane 2 vs 4). Notably, while the addition of exogenous RNF8 to the HeLa-G S100 extract in the absence of Tax had no effect on IKK (Fig 2E lane 4), exogenous RNF8 dramatically enhanced Tax-mediated activation of IKK (Fig 2E lane 3 vs 2). As expected, RNF8 addition to S100 extract prepared from RNF8_KD_ cells restored Tax-mediated IKK activation (Fig 2E lane 6 vs 7) while the truncated RNF8_345–485_ failed to do so (Fig 2E lane 9 vs 7), suggesting that the coiled-coil region and RING domain of RNF8 are not sufficient to effectively enhance Tax-mediated IKK activation.

### Ablation or over-expression of RNF8 drastically reduces or augments canonical NF-κB and JNK activation by Tax

**Fig 3:**
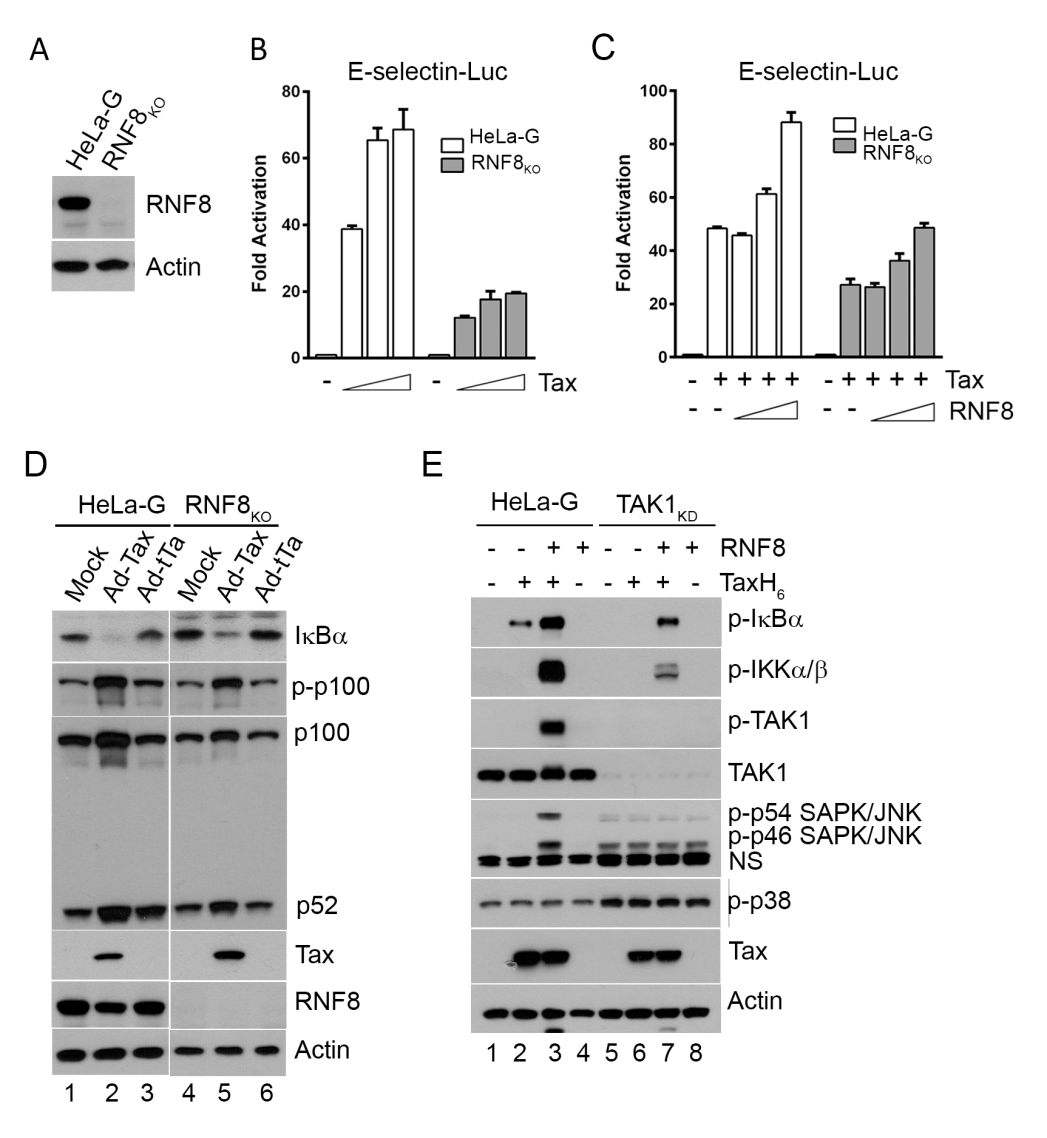
Tax and RNF8 potently activate IKK and the canonical NF-κB pathway. **(A)** HeLa-G cells with ablation of RNF8 were generated via CRISPR/Cas9 system and confirmed by immunoblotting. **(B)** Tax-mediated NF-κB activation is significantly diminished in RNF8_KO_ cells. HeLa-G or RNF8_KO_ cells (5x10^4^) were transiently co-transfected with E-Selectin-Luc (250 ng/ml) and increasing concentrations of Tax (0, 25, 50 and 100 ng/ml). Firefly luciferase activity and fold of activation for (B) and (C) below were measured and calculated as in Fig 1D. **(C)** RNF8 augmented and restored respectively Tax-mediated NF-κB activation in wild-type and RNF8_KO_ cells. As in (**B**), HeLa-G or RNF8_KO_ cells were co-transfected with E-Selectin-Luc (250 ng/ml), Tax (50 ng/ml), and increasing amounts of RNF8 (0, 50, 100, and 200 ng/ml). **(D)** RNF8 ablation does not affect non-canonical NF-κB activation by Tax. HeLa-G and RNF8_KO_ cells were mock transduced (lanes 1 & 4), or transduced with Ad-Tax (lanes 2 & 5) or Ad-tTa (for tet trans-activator) vector (lanes 3 & 6) for 48 hours. Both mock- and Ad-tTa-transduced cells were used as negative controls. Cells were harvested and immunoblotted for the indicated proteins. **(E)** Tax and RNF8 promote TAK1 and JNK activation *in vitro*. HeLa-G (lanes 1–4) or TAK1_KD_ (lanes 5–8) S100 extract was incubated with Tax and/or RNF8 as indicated at 30°C for 1 hour and immunoblotted for the indicated proteins. (NS: non-specific).

To confirm the crucial role of RNF8 in Tax-mediated IKK activation, an RNF8-null HeLa cell line (RNF8_KO_) was generated via the CRISPR/Cas9 system (Fig 3A). As anticipated, the loss of RNF8 reduced NF-κB activation by Tax to ∼1/3 to ∼1/2 of that in wild-type HeLa-G cells (Fig 3B shaded vs open bars). Co-transfection of Tax with increasing amounts of RNF8 in HeLa-G cells also augmented the already potent NF-κB activation by Tax in a dose-dependent manner (Fig 3C open bars). Finally, transfection of RNF8_KO_ cells with RNF8 DNA rescued Tax-induced NF-κB activation (Fig 3C shaded bars). Interestingly, in RNF8_KO_ cells, Tax continued to promote p100 phosphorylation and processing (Fig 3D p-p100 IB lanes 2 & 5 vs 1 & 4 and 3 & 6), suggesting that the non-canonical pathway is activated by Tax via an RNF8-independent mechanism. We note that the level of Rnf8 is reduced in Ad-Tax-transduced cells. This is likely due to the cell cycle arrest/senescence caused by Tax. In RNF8_KO_ cells, the extent of Tax-driven IκBα degradation was reduced but not abrogated (Fig 3D IκBα IB lanes 2 & 5 vs 1 & 4 and 3 & 6). Whether this is caused by IKKα and/or the K63-pUb conjugated to Tax (presumably by a different E3 ligase) remains to be determined.

In the S100 extract deficient in TAK1 (TAK1_KD_), IKK activation by Tax and RNF8 is greatly diminished (Fig 3E lanes 6 & 7 vs 2 & 3, p-IKKα/β blot). By contrast, in wild-type HeLa-G S100 extract, p-IκBα, p-IKKα/β, and p-TAK1 levels were dramatically enhanced by Tax and RNF8 in combination (Fig 3E lane 3), supporting the notion that Tax activates the canonical NF-κB pathway via RNF8, TAK1, and IKK. Most interestingly, significant p-JNK but not p-p38 kinase was readily detected in reactions containing exogenous Tax and RNF8 (Fig 3E lane 3), reminiscent of the constitutive activation of JNK in HTLV-1- or Tax-transformed cells reported previously [36]. We think this is likely due to Tax/RNF8-activated TAK1 effecting activation of MKK7 and its downstream target, JNK [37]. In aggregate, these data strongly suggest that the TAK1 activated by Tax/RNF8/Ubc13:Uev1A/Uev2 concurrently activated multiple downstream kinases including IKK and kinases upstream of JNK, possibly MKK7. For reasons unclear at present, a deficiency in TAK1 caused moderate p38 kinase and JNK activation as suggested by the presence of p-p54 and p-p46 SAPK/JNK and p-p38 in the S100 extract irrespective of Tax (Fig 3E bottom panel lanes 5–8).

### Tax causes increased cytoplasmic localization of RNF8

**Fig 4:**
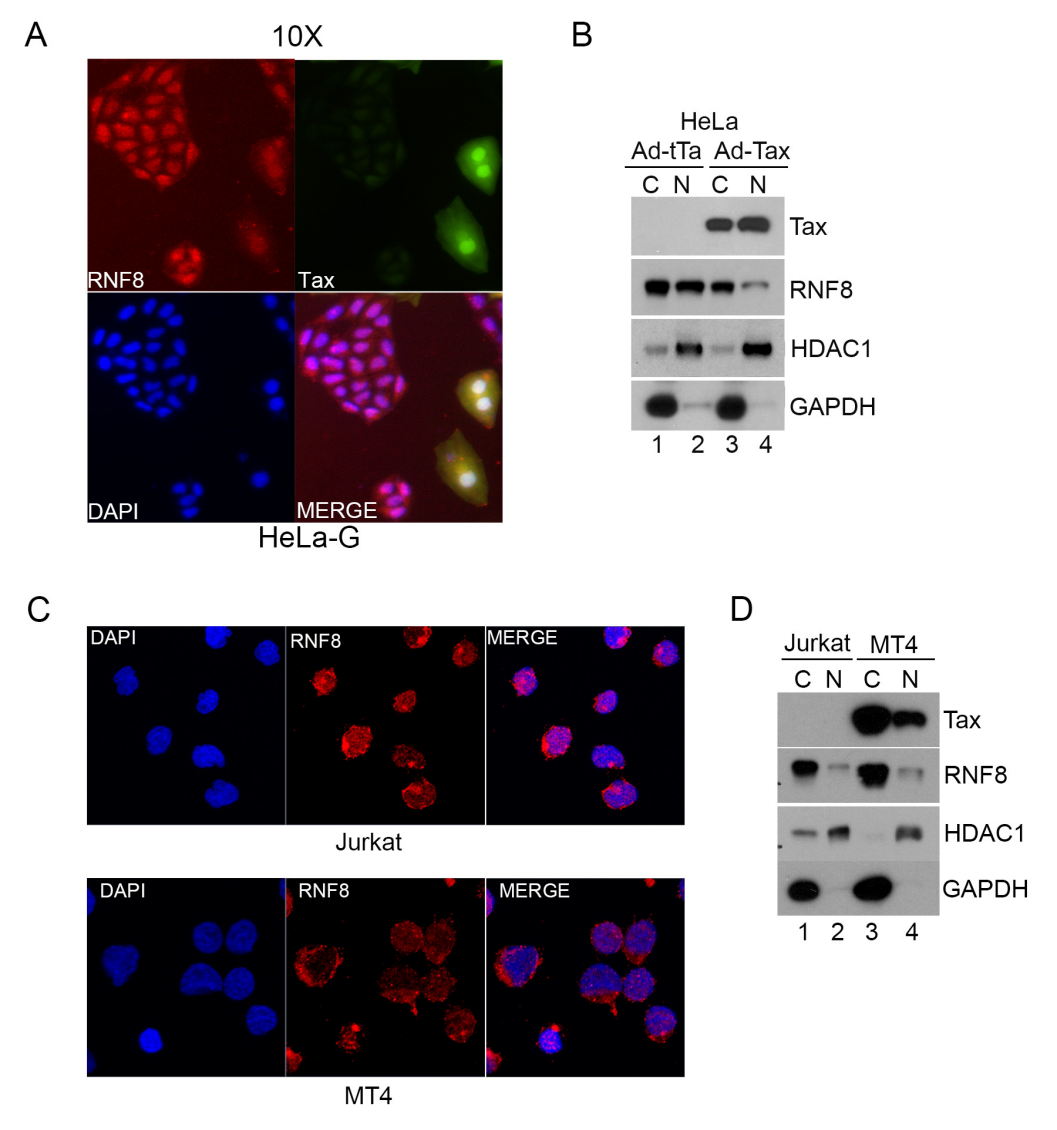
Tax redistributes RNF8 from the nucleus to the cytoplasm. **(A)** RNF8 is localized to the cytoplasm when Tax is present. HeLa-G cells were transduced with Ad-Tax at an MOI of 0.5 for 48 hours. HeLa-G cells contain a Tax-inducible GFP cassette and turn brightly green upon Tax expression as indicated by white arrows. The cells were then fixed, permeabilized and stained with an antibody for RNF8 (Red) and DAPI (Blue). **(B)** HeLa cells were transduced with Ad-Tax or Ad-tTa for 48 hours and fractionated following manufacturer’s protocol. The cytosolic and nuclear fractions were immunoblotted for the indicated proteins. **(C)** Much RNF8 is localized in the cytoplasm of Jurkat and HTLV-1-transformed MT4 T cell lines. Cells were fixed, permeabilized and stained with an antibody for RNF8 (Red) and DAPI (Blue). **(D)** Jurkat and MT4 cells were harvested and fractionated following manufacturer’s protocol. The cytosolic and nuclear fractions were immunoblotted for the indicated proteins. HDAC1 and GAPDH are used as nuclear and cytosolic protein control respectively for both **(B)** and **(D)**.

RNF8 is an E3 ligase that localizes primarily to the nucleus during interphase where it mediates DDR [30,38]. Since activation of TAK1 and IKK occurs in the cytoplasm, and Tax is known to shuttle between nuclear and cytoplasmic compartments [39], we asked if Tax might affect the cellular localization of RNF8. The Tax reporter cell line, HeLa-G [40], was transduced with Ad-Tax for 48 hours and the sub-cellular distribution of RNF8 determined by immunofluorescence. As indicated in Fig 4A, strong nuclear RNF8 signal (Red) could be readily detected in untransduced HeLa-G cells (GFP-). In contrast, in Tax-transduced cells (Fig 4A, upper right panel, GFP+), cytoplasmic RNF8 signal was increased (upper left panel). Subcellular fractionation of Tax-transduced HeLa-G cells also showed more RNF8 in the cytosolic than the nuclear fraction (Fig 4B lane 3 vs 4). HeLa-G cells transduced with the control Ad-tTa vector, by contrast, had an even distribution of RNF8 in both cytosolic and nuclear fractions (Fig 4B lane 1 vs 2). Further examination of HTLV-1-unrelated Jurkat and HTLV-1-transformed MT4 T cell lines by immunofluorescence (Fig 4C) and subcellular fractionation (Fig 4D) indicate that a significant fraction of RNF8 localizes to the cytosol of T cells, and Tax expression correlates with increased cytoplasmic distribution of RNF8. These results support the notion that Tax can redirect RNF8 to the cytoplasm for the activation of the canonical NF-κB pathway.

### Tax stimulates assembly of long K63-linked polyubiquitin by RNF8 and Ubc13:Uev1a (or Ubc13:Uev2) *in vitro* and *in vivo*

**Fig 5:**
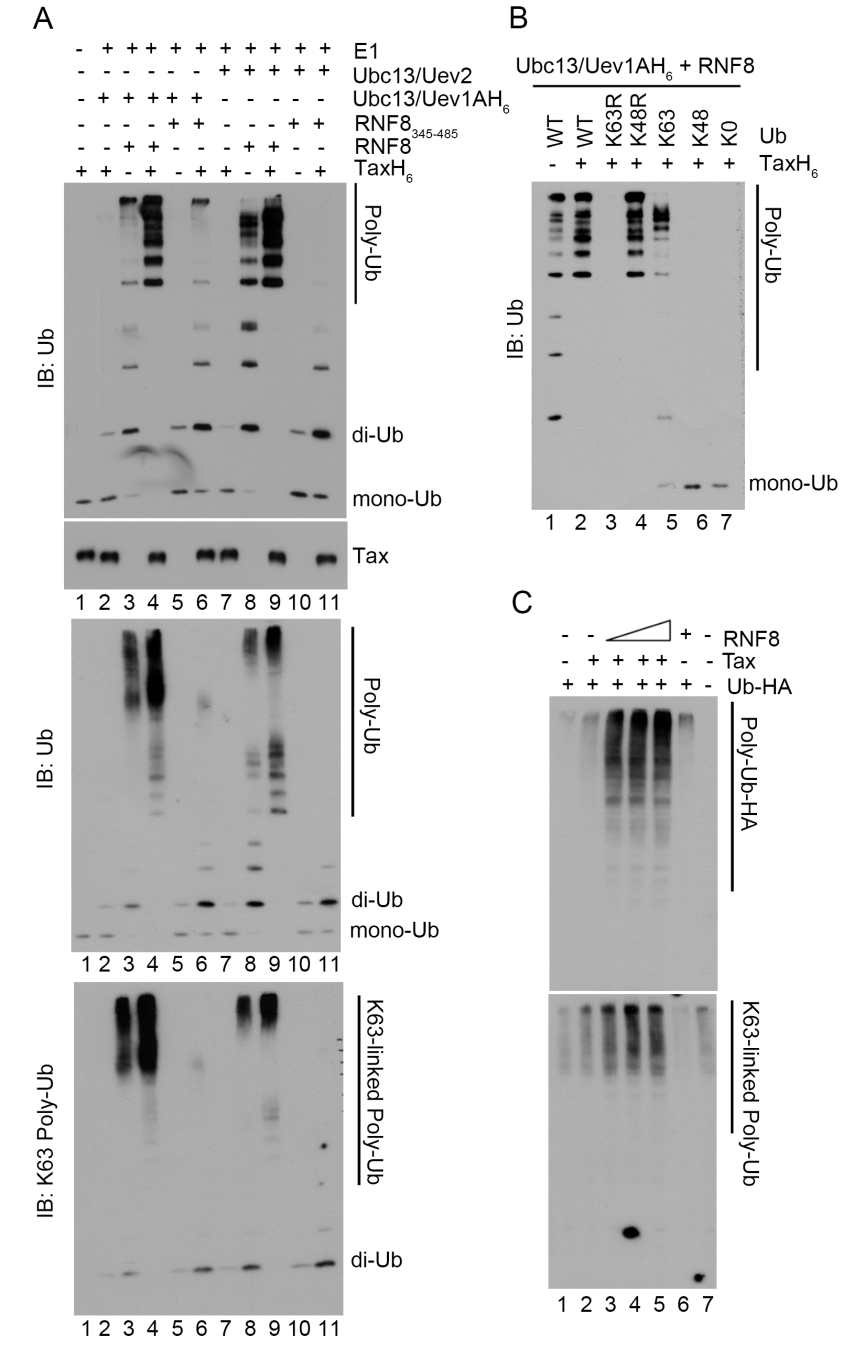
Tax stimulates RNF8 and Ubc13:Uev1A/2 to assemble long K63-pUb. **(A)** *In vitro* assembly of unanchored K63-pUb by RNF8 and RNF8_345–485_ in the presence or absence of Tax. Each ubiquitination reaction contained E1, ubiquitin, ATP, TaxH_6_, RNF8 or RNF8_345–485_, and Ubc13:Uev1A or Ubc13:Uev2 as indicated and incubated at 37^°^C for 4 hours. Reactions products were resolved in 15% (upper panel), 4–20% (middle panel), and 4–20% (bottom panel) polyacrylamide gels, respectively. They are probed with indicated antibodies (ubiquitin, upper and middle panels and K63-pUb, bottom panel). Reactions in lanes 2–6 and 7–11 contained Ubc13:Uev1A and Ubc13:Uev2 E2 enzyme complexes respectively. RNF8 and RNF8_345–485_ were used in reactions in lanes 3, 4, 8, 9 and 5, 6, 10, 11 respectively. **(B)** Polyubiquitin chain assembly by RNF8 and Ubc13:Uev1A in the presence of Tax requires ubiquitin molecules that contain lysine at amino acid residue 63. *In vitro* reactions were carried out as described in **(A)** with RNF8, Ubc13:Uev1A, ATP and E1, in the presence (lanes 2–7) or absence (lane 1) of TaxH_6_. Different variants of ubiquitin were used: wild-type ubiquitin (WT); K63R and K43R, mutants with lysine to arginine substitution at amino acid residue 63 and 48 respectively; K63 and K48, mutants with all lysine residues substituted by arginine except residue 63 and 48; and K0, a mutant with all lysine residues substituted by arginine. The protein concentration of the K63R ubiquitin mutant was based on the data sheet provided by the vender and was approximately ~1/4 of the other ubiquitin mutants, making it harder to detect by immunoblotting. **(C)** HeLa-G cells (10^5^) were co-transfected with Ub-HA (50 ng/ml), Tax (50 ng/ml), and/or increasing amounts of RNF8 (0, 50, 100, 200 ng/ml) as indicated for 48 hours. Cells were lysed with SDS sample buffer and immunoblotted for the indicated proteins.

Because K63-pUb plays a key role in IKK activation, we set out to determine if Tax directly impacts on K63-pUb chain assembly by RNF8 and Ubc13:Uev1a/Uev2. To this end, RNF8 and RNF8_345–485_ were incubated with E1, ubiquitin, ATP, Ubc13:Uev1A or Ubc13:Uev2, in the presence or absence of Tax *in vitro*. In the absence of Tax, a low level of polyubiquitin chain assembly by RNF8 and Ubc13:Uev1A or Ubc13:Uev2 could be detected *in vitro* (Fig 5A upper panel, lane 3 vs 2 for Ubc13:Uev1A; lane 8 vs 7 for Ubc13:Uev2), and Ubc13:Uev1a was more efficient than Ubc13:Uev2 in supporting polyubiquitin chain assembly (Fig 5A upper panel, lane 3 vs 8). Remarkably, the addition of Tax greatly stimulated polyubiquitin chain formation by RNF8 and Ubc13:Uev1A or Ubc13:Uev2 (Fig 5A upper panel, lane 4 vs 3; lane 9 vs 8). Tax also activated polyubiquitin chain assembly by RNF8_345–485_ (Fig 5A upper panel, lane 6 vs 5, for reactions containing Ubc13:Uev1A; lane 11 vs 10, Ubc13:Uev2), albeit the E3 ligase activity of RNF8_345–485_ and the extent of its activation by Tax were substantially lower compared to when full-length RNF8 was used (Fig 5A upper panel, for reactions with Ubc13:Uev1A: lane 6 vs 4 [with Tax], lane 5 vs 3 [without Tax], with Ubc13:Uev2: lane 11 vs 9 [with Tax], lane 10 vs 8 [without Tax]). The polyubiquitin chains assembled by RNF8 in the presence of Tax are of greater lengths compared to when RNF8_345–485_ was used, as revealed by analyzing the samples from the upper panel in a 4–20% polyacrylamide gradient gel (Fig 5A middle panel, lane 4 vs 6). As expected, RNF8_345–485_ failed to support IKK activation by Tax in the HeLa-G S100 extract (Fig 2E lane 9 vs 10), suggesting that the assembly of long K63-pUb chains is crucial for IKK activation. We also found Ubc13:Uev1A to be more robust than Ubc13:Uev2 for RNF8-driven long polyubiquitin chain assembly *in vitro* (Fig 5A middle panel, lane 4 vs 9), consistent with the reported activities of Ubc13:Uev1A and Ubc13:Uev2 [18].

Since a fraction of Tax is covalently modified by K63-pUb *in vivo*, we examined if Tax became polyubiquitinated by RNF8 and Ubc13 *in vitro*. The polyubiquitination reaction was carried out over a course of 8 hours. Even though Tax and RNF8 greatly stimulated IKK activity within 1 hour after their addition to the S100 extract, and Tax-driven K63-pUb chain assembly (by Ubc13:Uev1A and RNF8) *in vitro* peaked 4 hours into the *in vitro* reactions, only a hint of slower-migrating forms of Tax could be detected at 8 hours into the *in vitro* ubiquitin assembly reaction (S3 Fig). Thus the ubiquitination of Tax by RNF8 and Ubc13 *in vitro* does not correlate with IKK activation and is likely non-physiological. The polyubiquitination of Tax *in vivo* is likely carried out by an E3 ligase distinct from RNF8. Importantly, none of the other proteins (Uev1A, Uev2, and RNF8) in the *in vitro* reactions became polyubiquinated (S4 Fig). Thus the K63-pUb chain assembled by Tax/RNF8/Ubc13:Uev1A is unanchored and directly activates TAK1, and then IKK. These data agree with previous findings showing that free K63-pUb chains assembled by TRAF6 and Ubc13:Uev1A can activate TAK1 [41].

**Fig 6:**
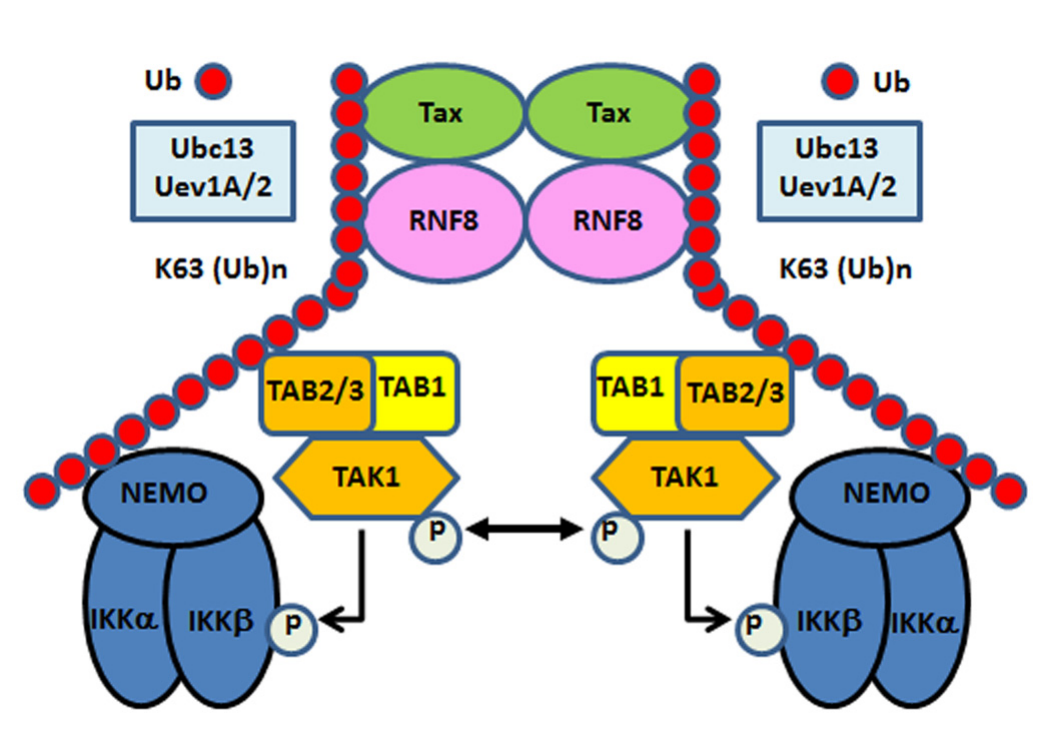
Tax hijacks RNF8 and Ubc13:Uev1A/Uev2 to activate IKK and the canonical NF-κB pathway. Tax interacts with and stimulates RNF8 and Ubc13:Uev1A/2 to assemble free K63-pUb chains, which serve as platforms for TAK1 and IKK to convene and activate the canonical NF-κB and other signaling pathways.

In agreement with the specificity of Ubc13 E2 enzyme complexes, the polyubiquitin chains assembled via RNF8 in the presence of Tax reacted to a K63-pUb-specific antibody (Fig 5A lower panel, lanes 3, 4, 6, 8, 9). Furthermore, only wild-type ubiquitin and ubiquitin mutants that contain lysine at amino acid residue 63 such as K48R and K63-only (all lysine residues except K63 mutated to arginine residues) (Fig 5B lanes 2, 4, and 5), but not those with altered K63 residue such as K63R, K48-only, and K0 (all lysine residues substituted with arginine residues) mutants (Fig 5B lanes 3, 6 and 7) supported Tax-induced polyubiquitin assembly *in vitro*. Finally, co-transfection of Tax with increasing amounts of RNF8 into HeLa cells stimulated in a dose-dependent manner the assembly of polyubiquitin chains that reacted with a K63-pUb-specific antibody (Fig 5C lanes 2–5 vs 1 & 7), while RNF8 alone had no effect (Fig 5C lane 6). Altogether, these results indicate that Tax usurps RNF8 and Ubc13:Uev1A/2 to assemble K63-pUb chains for the activation of TAK1, IKK, and other signaling pathways (summarized in Fig 6).

## Discussion

Many cancer viruses encode regulatory proteins (Epstein-Barr Virus LMP1, Kaposi Sarcoma Herpesvirus vFLIP, and HTLV-1 Tax) that activate IKK/NF-κB as a part of their oncogenic program, but the underlying molecular mechanisms remain incompletely understood. This hampers a clear understanding of the oncogenic processes and the development of treatment and therapeutic approaches. In this study, we have demonstrated *in vitro* and *in vivo* that Tax hijacks RNF8—a ubiquitin E3 ligase best known for ubiquitinating histones to signal DDR—to assemble K63-pUb chains for TAK1, IKK, and JNK activation (Fig 6). Depletion of RNF8 or constituents of the E2 conjugating enzymes, including Ubc13, Uev1A and Uev2, that RNF8 utilizes for K63-pUb assembly diminished while exogenous addition of RNF8 and Ubc13 greatly augmented Tax-induced IκBα phosphorylation in a cell-free system. These *in vitro* results were correlated with cell-based reporter assays. Importantly, using an *in vitro* system reconstituted with purified components, we have found that Tax dramatically activated RNF8 and Ubc13:Uev1a/Uev2 to assemble long and unanchored K63-pUb chains. Over-expression of RNF8 in the presence of Tax *in vivo* also led to a dose-dependent increase in K63-pUb synthesis. Activation of the non-canonical NF-κB pathway by Tax, however, is RNF8-independent and continues to occur in RNF8-null cells. These results demonstrate that Tax usurps cellular ubiquitination machinery to assemble K63-pUb chains for TAK1, IKK, and canonical NF-κB activation. Interestingly, Tax and RNF8 in combination also activated JNK phosphorylation *in vitro*. This is consistent with the role of TAK1 in regulating NF-κB-unrelated signaling pathways and provides an explanation for the pleiotropic effect of Tax.

Over the past few years, the theme of K63-pUb chains serving as signaling scaffolds has emerged (reviewed in [42–44]). Upon TNF-α or IL-1 stimulation, E3 ubiquitin ligases such as TRAF6, TRAF2/5, and cIAP1/2; and signaling molecules such as IRAK1 and RIP1 are recruited to the activated receptors where extensive K63 polyubiquitination takes place on TRAFs, RIP1, IRAK1 and other molecules [42–44]. K63-pUb chains then serve as signaling platforms for multiple kinases to convene and interact. A recent study has also shown that during IL-1β signaling, linear polyubiquitin (M1-pUb) assembled by a unique E3 ligase complex known as LUBAC (linear ubiquitin assembly complex) that consists of HOIP, HOIL, and Sharpin becomes covalently attached to K63-pUb to form K63/M1-pUb hybrid [45]. As NEMO, the regulatory subunit of IKK, has 100-fold higher binding affinity for M1-pUb than for K63-pUb, and TAB2/3, subunits of the holo-TAK1 complex, bind K63-pUb specifically, it is proposed that TAK1 and IKK respectively are recruited to K63-pUb and M1-pUb of the hybrid pUb such that TAK1 becomes auto-activated and signaling between TAK1 and IKK can occur [45]. Whether the assembly of K63-pUb triggers obligatory M1-pUb chain formation and whether M1-pUb chains are needed for Tax-mediated canonical NF-κB activation remain to be determined.

Both protein-linked and free unanchored K63-pUb chains have been reported to activate TAK1 and IKK complexes [41]. In the reconstituted system described herein, the K63-pUb chains assembled are free and unconjugated to protein factors used in the assay including Tax, RNF8 and Ubc13. Tax has been shown to be modified by ubiquitination and sumoylation [46,47]. Since Tax is not polyubiquitinated by RNF8 *in vitro* (S3 Fig), its polyubiquitination may require other E3 ligases.

Other cellular factors including RNF4 and NRP/Optineurin have been reported to play a role in Tax-mediated NF-κB activation [48,49]. We have found that under the condition of our experiment, RNF4 moderated NF-κB activation by Tax (S5 Fig). This may be related to the K48-linked polyubiquitination and degradation of Tax promoted by RNF4 [50]. Whether and how NRP/Optineurin impacts the Tax-RNF8 signaling axis remains to be determined.

RNF8 is involved in the early signaling events of the DNA double-stranded break repair pathway. Via its N-terminal FHA domain, RNF8 is targeted to the ataxia telangiecstasia mutated (ATM)-phosphorylated mediator of DNA damage checkpoint 1 (MDC1) that binds to phospho-histone H2 variant H2ax (γ-H2ax) accumulating at the site of DNA double-strand breaks where RNF8 promotes K63 polyubiquitination of histones H2a, H2b and γ-H2ax. This then leads to the recruitment of another E3 ligase, RNF168. The extensive K63-polyubiqutination of histones by RNF8 and RNF168 facilitates the recruitment of the p53-binding protein 1 (53BP1) and the breast cancer susceptibility protein (BRCA1) for DDR [24,29,30,51]. RNF8 has also been shown recently to localize to sites of cell division where it stimulate K63 polyubiquitination of septin 7 for cytokinesis [31]. Tax-RNF8 interaction therefore may play a role in DDR [52] and cytokinesis [53] defects induced by Tax. Since RNF8 ablation did not cause overt cytological abnormalities in HeLa-G cells, it appears that the cytopathic effects of Tax cannot be explained based solely on RNF8 sequestration. Whether the over-production of mislocalized K63-pUb chains as stimulated by Tax may sequester cellular factors crucial for DDR and cytokinesis is currently under investigation. As the regulation of RNF8 and related RING-domain E3 ligases is poorly understood, elucidating how Tax interacts with and activates RNF8 will provide critical insight into how this class of E3 ligases is regulated. Finally, a clear understanding of how Tax impacts cellular signaling will shed light on the development of ATL and facilitate the design of therapeutic approaches.

## Materials and Methods

### Derivation of knockdown cell lines

Knockdown of each of the Ubc13, Uev2, Uev1A and RNF8 genes in a Tax reporter HeLa/18x21-EGFP (HeLa-G, derived in the lab) cell line was performed as previously described [54,55]. The sequences targeted for each gene are listed in S1 Table). Stable cell clones with knockdown of each gene were isolated by limiting dilution and gene silencing validated by immunoblotting.

### Derivation of RNF8 knockout cell line

RNF8 knock out cell line was generated using the CRISPR/Cas9 system as described in [56]. Briefly, two complementary oligonucleotides, CACCGTCACAGGAGACCGCGCCGG and AAACCCGGCGCGGTCTCCTGTGAC, corresponding to the 5’ coding region of human RNF8 were synthesized. After annealing, the double-stranded DNA fragment was cloned into the CRISPR/Cas9 vector pX330 (Addgene) that had been cut with BbsI. The recombinant plasmid is transfected into HeLa cells by electroporation. Stable clones with RNF8 knockout were screened by immunoblotting after limiting dilution.

### Cell culture

HEK293T (ATCC), HeLa-G, and HeLa-G knockdown and knockout cell lines were cultured in DMEM supplemented with 10% fetal bovine serum, L-glutamine, 100U/ml penicillin and streptomycin and maintained in 5% CO_2_ at 37^°^C. Jurkat T cells (ATCC) were grown in RPMI with the same supplements.

### Immunoblotting

Cells were harvested and lysed in lysis buffer (Cell Signaling). Routinely, a total of 10–20 μg of proteins is loaded per sample. HTLV-1 Tax hybridoma monoclonal antibody 4C5 was as previously described [53]. Ubc13, IKKα, IKKβ, TAK1, p100/p52, p-IκBα, p-IKKα/β, p-TAK1, p-JNK/SAPK, p-p38, p-p100 antibodies were from Cell Signaling Technology. IκBα, RNF8, HDAC1, GAPDH, β-actin, HA and ubiquitin antibodies were from Santa Cruz Biotechnology. Uev2 antibody was from Abcam. K63 ubiquitin antibody was from eBioscience. Poly-Histidine antibody was from Sigma-Aldrich.

### S-tagged pull-down assay

HEK293T cells were transfected with a *PiggyBac* transposon-based plasmid (a kind gift from Dr. Pentao Liu [57]) for Tax-S-Tag. Cells were harvested 48 hours later and lysed with lysis buffer containing a deubiquitinase inhibitor, PR619 (Life Sensors). Cleared cell lysate was then incubated with S-protein agarose beads (Novagen) at 4^°^C overnight. The beads were washed three times with lysis buffer and the protein was then eluted in equal volume of 2X Laemmli Sample Buffer (Sigma-Aldrich). The eluted protein was heated to 95^°^C for 10 minutes for immunoblotting.

### DNA transfection and luciferase reporter assay

HeLa-G cells were co-transfected with E-Selectin luciferase NF-κB reporter plasmid and BC12-Tax, pcDNA-RNF8 and/or RNF4-mCherry by lipofection (using Promega FuGENE transfection reagent) for 48 hours. DNA transfection was typically carried out in triplicate in a 24-well plate seeded the night before with 5x10^4^ HeLa-G cells per well in 0.5 ml DMEM supplemented with 10% fetal bovine serum. Each transfection contained E-Selectin luciferase (250 ng/ml), BC-12 Tax (50 ng/ml, unless otherwise indicated). The final DNA amount per well is adjusted to 1 μg/ml using pcDNA plasmid. The Luciferase Reporter Assay was performed following manufacturer’s protocol (Promega). The luminescent signals were detected using Glomax Multi Detection System (Promega). To detect K63-pUb formation as a function of Tax and RNF8 expression, 1x10^5^ cells/well were grown in 1 ml medium in a 12-well plate and transfected with the indicated amounts of DNA.

### Preparation of cytosolic extract (S100)

S100 was prepared as in [20]. Briefly, 1.5 x 10^8^ cells were re-suspended in 500 μL of a hypotonic buffer and homogenized using a Dounce homogenizer. Cleared supernatant (S100) was collected after ultracentrifugation at 100,000 g for 1 hour.

### Cell-free IKK activation assay

Cell-free assay was performed as in [17]. The S100 cytosolic extract (~20 μl at a protein concentration of at least 10 mg/ml) was incubated with 0.5 μM recombinant TaxH_6_, 0.35 μM recombinant RNF8, 0.5 μM Ubc13/Uev2 (Life Sensors) and/or 0.5 μM Ubc13H_6_/Uev1A (Boston Biochem) as indicated in an ATP-containing buffer at 30^°^C for 1 hour. The reactions were quenched by adding 2X Laemmli Sample Buffer (Sigma Aldrich) and heated to 95^°^C for 10 minutes for analysis by immunoblotting.

### *In vitro* ubiquitination assay

Ubiquitination assay was carried out as in [27]. A ubiquitination reaction typically contains 25 μM wild-type or mutant ubiquitin (Life Sensors), 0.1 μM human E1 (Life Sensors), 0.4 μM His_6_-Ubc13/Uev1A (Boston Biochem) or Ubc13/Uev2 (Life Sensors), 0.75 μM recombinant E3 ligase (RNF8 or RNF8_345-485_) and/or 0.5 μM recombinant Tax in 20–25 μL of ubiquitination buffer (20 mM HEPES pH 6.8, 200 mM NaCl, 2.5 mM MgSO_4_, 10 μM ZnSO_4_, 0.1 mM DTT, 2 mM ATP) and incubated at 37°C for 4 hours or the indicated times. The reactions were then quenched and immunoblotted as above.

### Immunofluorescence

HeLa-G cells were plated on chamber slides and transduced with Ad-Tax (MOI of 0.5) for 48 hours. T cells were plated on poly-L-lysine-coated cover slips for 10 minutes. They were then fixed with 4% paraformaldehyde and permeabilized with 0.2% Triton X-100. Cells were immunostained overnight with RNF8 primary antibody (Santa Cruz Biotechnology) followed by Alexa Fluor 568 secondary antibody (Invitrogen). The slides were then mounted in Dako Fluorescence Mounting Medium (Agilent Technologies) and set at room temperature for 1 hour in the dark. Images were captured using an Olympus IX81F fluorescence microscope or a Pascal confocal microscope.

### Subcellular fractionation

HeLa-G cells were transduced with Ad-Tax or Ad-tTa (MOI of 20) for 48 hours. Cells were harvested and immediately fractionated using a Nuclear and Cytoplasmic Extraction kit by Thermo Scientific. The fractions were then immunoblotted for the indicated proteins.

### Protein expression and purification

Hexahistidine-tagged Tax protein (TaxH_6_) was expressed and purified as previously described [58]. Recombinant GST-tagged human RNF8_345-485_ [27] was expressed in *E*. *coli* BL21 DE3 after IPTG induction. Cell pellet obtained from 2 liters of culture was re-suspended in lysis buffer (50 mM Tris pH 8.0, 150 mM NaCl, 1 mM DTT and protease inhibitor cocktail) and lysed using a French Press. The lysate was cleared by centrifugation at 43,000 rcf for 30 minutes. Cleared lysate was incubated with 200–300 μL 50% glutathione agarose bead slurry at 4^°^C overnight and the beads were washed with lysis buffer. GST-RNF8_345-485_ was then cleaved on the beads with PreScission Protease (GE HealthCare) to release RNF8_345-485_. Recombinant GST-tagged full-length human RNF8 (GST-RNF8) was expressed in Sf9 cells (from G. Dveksler) after infection with a baculovirus vector (a kind gift from Dr. Titia Sixma [28]). Cell pellet was re-suspended in lysis buffer (30 mM HEPES pH 8.0, 250 mM NaCl, 10% glycerol, 1 μM ZnCl_2_, 1 mM TCEP, and protease cocktail) and lysed over a salt-ice bath by sonication (microtip, 60% duty cycle, 15 seconds on and 30 seconds off for 5 minutes). The lysate was cleared by centrifugation at 43,000 rcf for 30 minutes. Cleared lysate was incubated with glutathione agarose beads at 4^°^C for 2 hours and the beads were washed with lysis buffer. GST-RNF8 was eluted with 10 mM reduced glutathione and cleaved with PreScission Protease. The GST moiety was removed by passing the reaction mixture through glutathione agarose beads. Recombinant hexa-histidine-tagged Tax (TaxHis_6_) was expressed in *E*. *coli* BL21 DE3 and purified using a HisTrap nickel column (GE Healthcare) with an FPLC as previously described [58] except cell lysis was carried out using a French press. The purified TaxHis_6_ was dialyzed (20 mM Hepes pH 7.9, 100 mM KCl, 0.5 mM DTT, 0.2 mM EDTA, 0.5 mM PMSF, 20% glycerol) and stored frozen at -80^°^C.

### RNA extraction and real-time quantitative RT-PCR

Total mRNA was isolated from HeLa-G and Uev1A knockdown cell clones using TRIzol Reagent (Ambion) according to manufacturer's instructions. Turbo DNA-free kit (Ambion) was used to remove contaminating genomic DNA. Complementary DNA (cDNA) was then synthesized using iScript reverse transcription super mix (Biorad). Real-time PCR was performed using the cDNA as templates with Uev1A specific primers purchased from BioRad or β-actin specific primers (S2 Table), and LightCycler DNA SYBR Green I master mix (Roche Applied Science) in a LightCycler thermal cycler (Roche Diagnostics). The mRNA level in each sample was normalized to that of the β-actin mRNA. Relative mRNA levels were calculated using the 2−ΔCt method [59].

## Supporting Information

S1 Table shRNA clones and their target sequences. (DOCX)

S2 Table Primers used for real-time qPCR. (DOCX)

S1 Fig Uev1A mRNA expression in wild-type HeLa and Uev1A knockdown cell line. Uev1A mRNA was obtained from HeLa-G and Uev1A_KD_ cells and measured via real-time PCR. Fold of change was calculated using the 2−ΔCt method.
(TIF)

S2 Fig Ubc13-containing E2 conjugating enzyme complexes enhanced Tax-mediated IKK activation in Jurkat S100 extract. The Jurkat cytosolic S100 extract (lane 1) was incubated with recombinant TaxH_6_ alone (lanes 2), Ubc13, Ubc1 and Uev2, Ubc13/Uev1AH_6_ or UbcH5b/c with (lanes 6–8, 11–12) or without TaxH_6_ (lanes 3–5, 9–10) as indicated at 30°C for 1 hour. IKK activation was detected by immunoblotting with anti-p-IκBα and p-IKKα/β. An immunoblot of total IκBα indicates a quantitative conversion of IκBα to the slower-migrating phosphorylated form in reactions supplemented with Tax, Ubc13, Uev1a, and Uev2 (lanes 6–8). We note that Ubc13/Uev1AH_6_ addition to Jurkat extract did not have the same stimulatory effect as in HeLa extract. This is likely due to the relative abundance of Ubc13, Uev1A, and Uev2 in Jurkat versus HeLa cells. When the amount of a given E2 enzyme complex is not limiting, the exogenous addition of that enzyme will have less of a stimulatory effect.
(TIF)

S3 Fig Tax is not polyubiquitinated by RNF8. *In vitro* polyubiquitination reactions containing TaxH_6_, RNF8, Ubc13:Uev1A, ATP and E1 were carried out as described in Fig 5A. Reactions were incubated for 2, 4, and 8 hours as indicated and immunoblotted for Tax.
(TIF)

S4 Fig Uev1A, Uev2, RNF8 and Tax are not poly-ubiquitinated in *in vitro* polyubiquitination assay. *In vitro* polyubiquitination reactions containing TaxH_6_, RNF8, Ubc13:Uev1A or Ubc13:Uev2, E1 and ATP were carried out as described in Fig 5A. Reactions were incubated for 4 hours and immunoblotted for the indicated proteins.
(TIF)

S5 Fig RNF4 abated Tax-mediated NF-κB activation. HeLa-G cells (5x10^4^) were transiently co-transfected with E-Selectin-Luc (250 ng/ml), Tax (50ng/ml), RNF8 (250 ng/ml) and/or RNF4 (250 ng/ml) for 48 hours. Firefly luciferase activity and fold of activation were measured and calculated as in Fig 1D.
(TIF)
