# Supplementary material for: HTLV-1 Tax Stimulates Ubiquitin E3 Ligase, Ring Finger Protein 8, to Assemble Lysine 63-Linked Polyubiquitin Chains for TAK1 and IKK Activation
Source: PLoS Pathog. 2015 Aug 18;11(8):e1005102. doi: 10.1371/journal.ppat.1005102 (PMC4540474; doi:10.1371/journal.ppat.1005102)
Supplement: S1 Table — (DOCX) [file ppat.1005102.s001.docx]

**Supplemental Table 1: shRNA clones and their target sequences**

| **Gene symbol** | **Clone ID** | **NM ID** | **Target sequence** |
| --- | --- | --- | --- |
| Ubc13 (UBE2N) | TRCN0000007213 | NM_003348 | GCTGAGGCATTTGTGAGTCTT |
| Ubc13 (UBE2N) | TRCN0000007214 | NM_003348 | GCCTTGTTAAGTGCTCCCAAT |
| Ubc13 (UBE2N) | TRCN0000007215 | NM_003348 | CCATAGAAACAGCTAGAGCAT |
| Ubc13 (UBE2N) | TRCN0000007216 | NM_003348 | CCTTCCAGAAGAATACCCAAT |
| Ubc13 (UBE2N) | TRCN0000007217 | NM_003348 | CTAGGCTATATGCCATGAATA |
| Uev2 (UBE2V2) | TRCN0000004034 | NM_003350 | CAAGGTGGACAGGCATGATTA |
| Uev2 (UBE2V2) | TRCN0000004035 | NM_003350 | CAGAAGGACAAACATACAACA |
| Uev2 (UBE2V2) | TRCN0000004036 | NM_003350 | GTCTTAAATCAACAACCTTCT |
| Uev2 (UBE2V2) | TRCN0000004037 | NM_003350 | GCTCCTCCGTCAGTTAGATTT |
| Uev2 (UBE2V2) | TRCN0000284952 | NM_003350 | TCAAGAGCTAAGACGTCTAAT |
| Uev2 (UBE2V2) | TRCN0000284956 | NM_003350 | GGGCCACCAAGGACAAATTAT |
| Uev2 (UBE2V2) | TRCN0000273330 | NM_003350 | CAAGGTGGACAGGCATGATTA |
| Uev2 (UBE2V2) | TRCN0000273329 | NM_003350 | GCTCCTCCGTCAGTTAGATTT |
| Uev2 (UBE2V2) | TRCN0000273328 | NM_003350 | GTCTTAAATCAACAACCTTCT |
| Uev1A (UBE2V1) | TRCN0000033704 | NM_021988 | CCCTGGTTTCTTTAAGTCTTA |
| Uev1A (UBE2V1) | TRCN0000033705 | NM_021988 | GCAAACTGAGTGATGAAGGAA |
| Uev1A (UBE2V1) | TRCN0000033706 | NM_021988 | CCAAGAGCCATATCAGTGCTA |
| Uev1A (UBE2V1) | TRCN0000033707 | NM_021988 | AGGACAGTGTTACAGCAATTA |
| Uev1A (UBE2V1) | TRCN0000033708 | NM_021988 | CGCCTAATGATGTCTAAAGAA |
| RNF8 | TRCN0000003437 | NM_003958 | GCCTGAATGTACTATGTTTAA |
| RNF8 | TRCN0000003438 | NM_003958 | TGGAGCAACTAGAGAAGACTT |
| RNF8 | TRCN0000003439 | NM_003958 | GAAGCCGTTATGAATGTGAAA |
| RNF8 | TRCN0000003440 | NM_003958 | CAAAGAATTAGAGCAGACCAA |
| RNF8 | TRCN0000003441 | NM_003958 | CCAAAGAATGACCAAATGATA |
| TAK1 | TRCN0000001554 | NM_145332 | GCAGTGATTCTTGGATTGTTT |
| TAK1 | TRCN0000001555 | NM_145332 | CCCGTGTGAACCATCCTAATA |
| TAK1 | TRCN0000001556 | NM_145332 | CAGTGTGTCTTGTGATGGAAT |
| TAK1 | TRCN0000001557 | NM_145332 | GACACACATGACCAATAACAA |
| TAK1 | TRCN0000001558 | NM_145332 | TCCTGCCACAAATGATACTAT |
