# Supplementary material for: HTLV-1 Tax Stimulates Ubiquitin E3 Ligase, Ring Finger Protein 8, to Assemble Lysine 63-Linked Polyubiquitin Chains for TAK1 and IKK Activation
Source: PLoS Pathog. 2015 Aug 18;11(8):e1005102. doi: 10.1371/journal.ppat.1005102 (PMC4540474; doi:10.1371/journal.ppat.1005102)
Supplement: S2 Table — (DOCX) [file ppat.1005102.s002.docx]

**Supplemental Table 2: Primers used for real-time qPCR**

| β-actin-F | 5'- TGAGCTGCGTGTGGCTCC |
| --- | --- |
| β-actin-R | 5'- GGCATGGGGGAGGGCATACC |
