# Supplementary figures and images for: HTLV-1 Tax Stimulates Ubiquitin E3 Ligase, Ring Finger Protein 8, to Assemble Lysine 63-Linked Polyubiquitin Chains for TAK1 and IKK Activation
Source: PLoS Pathog. 2015 Aug 18;11(8):e1005102. doi: 10.1371/journal.ppat.1005102 (PMC4540474; doi:10.1371/journal.ppat.1005102)

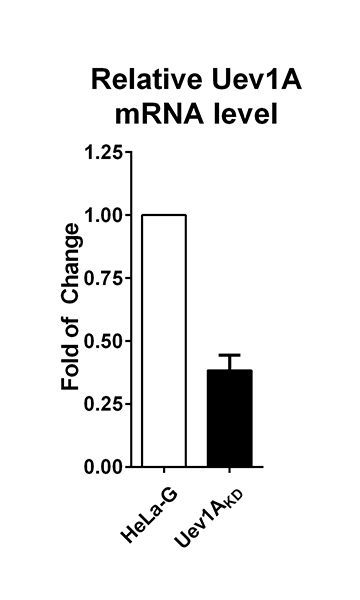

Supplement: S1 Fig — Uev1A mRNA was obtained from HeLa-G and Uev1AKD cells and measured via real-time PCR. Fold of change was calculated using the 2−ΔCt method. (TIF) [file ppat.1005102.s003.tif]

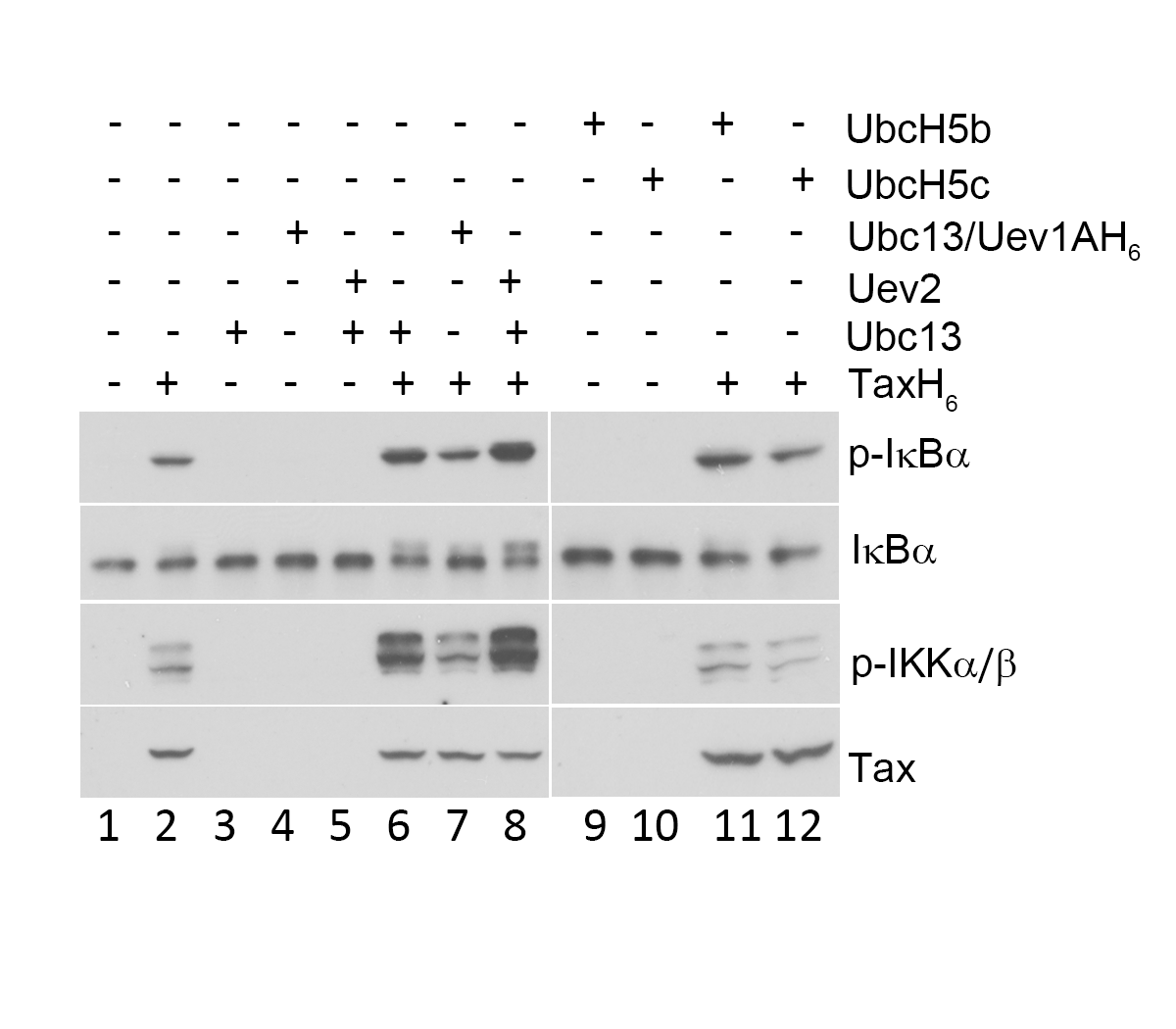

Supplement: S2 Fig — The Jurkat cytosolic S100 extract (lane 1) was incubated with recombinant TaxH6 alone (lanes 2), Ubc13, Ubc1 and Uev2, Ubc13/Uev1AH6 or UbcH5b/c with (lanes 6–8, 11–12) or without TaxH6 (lanes 3–5, 9–10) as indicated at 30°C for 1 hour. IKK activation was detected by immunoblotting with anti-p-IκBα and p-IKKα/β. An immunoblot of total IκBα indicates a quantitative conversion of IκBα to the slower-migrating phosphorylated form in reactions supplemented with Tax, Ubc13, Uev1a, and Uev2 (lanes 6–8). We note that Ubc13/Uev1AH6 addition to Jurkat extract did not have the same stimulatory effect as in HeLa extract. This is likely due to the relative abundance of Ubc13, Uev1A, and Uev2 in Jurkat versus HeLa cells. When the amount of a given E2 enzyme complex is not limiting, the exogenous addition of that enzyme will have less of a stimulatory effect. (TIF) [file ppat.1005102.s004.tif]

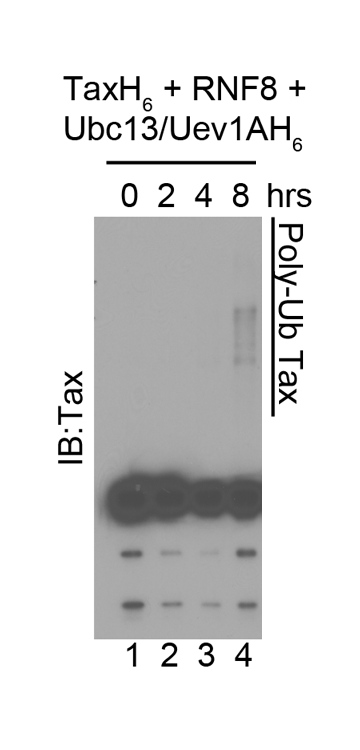

Supplement: S3 Fig — In vitro polyubiquitination reactions containing TaxH6, RNF8, Ubc13:Uev1A, ATP and E1 were carried out as described in Fig 5A. Reactions were incubated for 2, 4, and 8 hours as indicated and immunoblotted for Tax. (TIF) [file ppat.1005102.s005.tif]

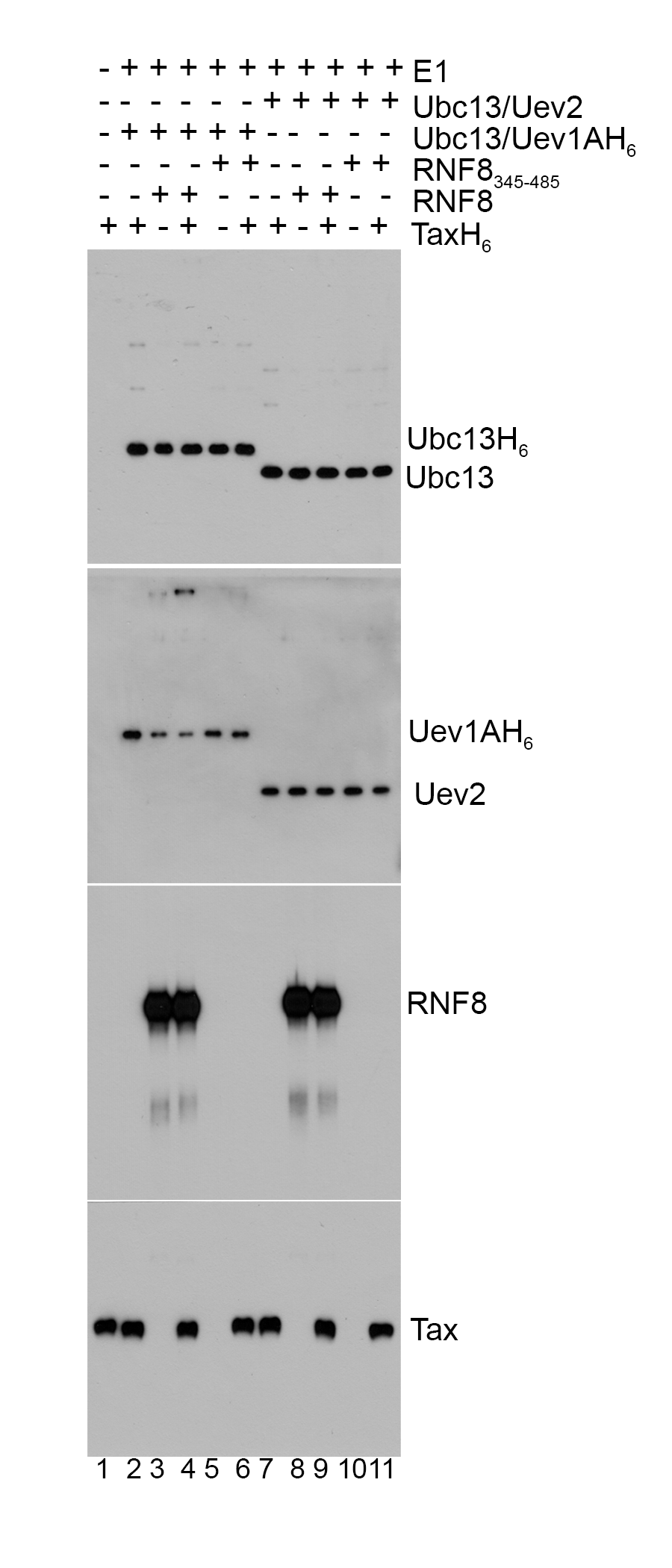

Supplement: S4 Fig — In vitro polyubiquitination reactions containing TaxH6, RNF8, Ubc13:Uev1A or Ubc13:Uev2, E1 and ATP were carried out as described in Fig 5A. Reactions were incubated for 4 hours and immunoblotted for the indicated proteins. (TIF) [file ppat.1005102.s006.tif]

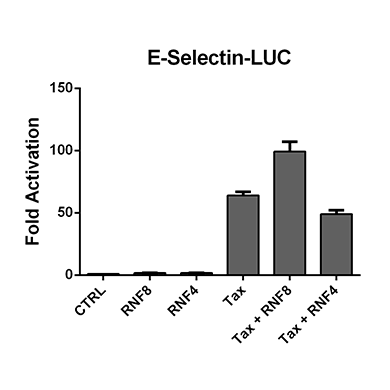

Supplement: S5 Fig — HeLa-G cells (5x10^4^) were transiently co-transfected with E-Selectin-Luc (250 ng/ml), Tax (50ng/ml), RNF8 (250 ng/ml) and/or RNF4 (250 ng/ml) for 48 hours. Firefly luciferase activity and fold of activation were measured and calculated as in Fig 1D. (TIF) [file ppat.1005102.s007.tif]
